# The Use of Copper in Syphilis

**Published:** 1903-11-07

**Authors:** 


### THE USE OF COPPER IN SYPHILIS.

As the result of long observation and experience Dr. A. F. Price[1] has concluded that copper is of considerable value as an addition to the generally accepted methods of treating syphilis, and that it possesses some peculiarities that are of great interest and command attention. The most important of these is an intolerance of the copper salts, which, when it exists, is, Dr. Price believes, characteristic of long-standing syphilis. In commencing to treat a case of old syphilis he usually gives a tablet containing $\frac{1}{3200}$ gr. of copper arsenite, and frequently this small dose cannot be borne, and will produce prostration and almost collapse. So far as he is aware there is no other disease in which this sensitiveness to copper exists, and he therefore suggests that the drug may be a valuable diagnostic aid in doubtful cases. However, by persevering in the administration of small doses toleration may be established and the quantity of copper given daily may be gradually increased. As soon as a dose of $\frac{1}{600}$ gr. of the arsenite can be tolerated, Dr. Price substitutes copper sulphate. If the drug be used over a long period of time in this way it is capable of producing marked amelioration of the symptoms which are characteristic of the so-called parasyphilitic affections—locomotor ataxia and general paralysis. In none of the many cases of tabes dorsalis of syphilitic origin which have been under Dr. Price's care has the copper treatment failed to be of benefit.

One precaution is necessary, and this is to avoid causing neuritis. Copper has a decided tendency to produce this complication, and it is therefore advisable not to continue the administration of the drug without intermission, and it is also beneficial to give the patient laxatives and salines while the copper treatment is being employed. Although copper is of special value in the treatment of long-standing syphilis, Dr. Price is of the opinion that the drug may be advantageously employed in all stages of the disease.

In connection with Dr. Price's remarks on the value of copper salts in the treatment of tabes dorsalis and general paralysis it is interesting to note that at one time copper had a considerable reputation in the treatment of affections of the central nervous system. In view of these facts the drug would certainly seem to be worthy of an extended trial in the parasyphilitic affections where present methods of treatment are so unsatisfactory.

[1] Medical Record, Oct. 10, 1903.

